# A systematic review of penile prosthesis infection and meta-analysis of diabetes mellitus role

**DOI:** 10.1186/s12894-020-00730-2

**Published:** 2021-03-10

**Authors:** Lucas Mira Gon, Caio César Citatini de Campos, Brunno Raphael Iamashita Voris, Luís Augusto Passeri, Adriano Fregonesi, Cássio Luís Zanettini Riccetto

**Affiliations:** Division of Urology of Department of Surgery, Faculty of Medical Sciences, Hospital de Clinicas, University of Campinas – UNICAMP, Rua Vital Brazil, 250, Campinas, SP 13083-590 Brazil

**Keywords:** Penile prosthesis, Penile implants, Infection, Review, Meta-analysis, Diabetes mellitus

## Abstract

**Background:**

Infection is the most feared complication of a penile prosthesis. Diabetes mellitus (DM) is widely known to increase the risk of several infections, but its role in the penile prosthesis is still controversial. This systematic review aims to show the contemporary scenario of penile prosthesis infection and present a meta-analysis about DM contribution to penile prosthesis infection.

**Methods:**

The review was performed with no language or time limitation, including ten databases. The included articles were about the male population who received a penile prosthesis with no model restriction, with a minimum follow up of 1 year, and outcomes adequately reported.

**Results:**

The mean infection incidence of penile prosthesis ranged from 0.33 to 11.4%. In early 2000, the general incidence of infection was 3 to 5%, then, the introduction of coated materials decreased it to 0.3 to 2.7%. The meta-analysis showed that diabetes mellitus is related to an increased risk of penile prosthesis infection with an *odds ratio *of 1.53 (95% CI 1.15–2.04).

**Conclusions:**

Penile prosthesis infection decreased in the last decades but remains a significant cause of reoperation, and it is related to lower prosthesis survival. Meta-analysis concludes that diabetes mellitus is related to a higher risk of penile prosthesis infection.

## Background

The penile prosthesis was introduced in the 1970s and remained the most effective treatment to erectile dysfunction refractory to oral and injectable drugs [1, 2]. In the last 40 years, several improvements in materials and surgical techniques led to high satisfaction rates of 80 to 90% [3, 4]. However, complications do exist and range from 7 to 20% [5], mostly related to mechanical malfunction, infection, and erosion. The estimated cost of penile prosthesis removal is about 10 thousand dollars, which is six-fold higher than the initial implantation [6].

Surgical site infection is the most feared complication. It causes pain, local abscess, and even sepsis that requires prompt hospitalization and reoperation [7]. The prosthesis removal leads to fibrosis of the cavernosum corpus and reduction of penile length and girth, making a new prosthesis insertion much more difficult [8].

Diabetes mellitus (DM) impairs microcirculation and causes neuropathy, and approximately 50% of diabetic patients have some degree of erectile dysfunction. The corpus cavernosum of diabetic patients are less responsive to relaxation due to the superoxide radicals production, impairing nitrous oxide and cyclic-GMP production. Thus, diabetic patients are less responsive to oral therapy [9]. Patients with diabetes are more prone to infection because of leucocyte dysfunction and microangiopathy. There is evidence of a three-fold higher risk of penile prosthesis infection in DM compared to non-diabetic patients. However, other studies show no difference, and there is still controversy about whether DM increases the risk of penile prosthesis infection [10, 11].

Several device improvements reduced mechanical failures of penile prosthesis, but infection remained an important cause of reoperation. Therefore, the efforts were directed to reduce infection in the past years [8, 12]. This study aims to gather information about penile prosthesis evolution regarding infections and to present a meta-analysis of diabetes mellitus contribution to penile prosthesis infection.

## Methods

This review was performed with no language or time limitation, to gather all available data about penile prosthesis, in ten databases: Medline, PubMed, LILACS, IBECS, MEdCarib, CINAHL, Scopus, Web of Science, Embase and Cochrane Library. The search strategy included the terms: “*penile prosthesis*” or “*penile implantation*” and “*postoperative complications*” or “*prosthesis-related infections*” or “*treatment outcome*”. It followed the PRISMA statement, was registered at PROSPERO with number CRD 42019117734, and had no founding resources.

All the articles had the title and abstract evaluated by two independent authors who selected relevant studies blinded from each other. A third and more experienced author resolved conflicting selection. The included articles were about the male population who received a penile prosthesis with no model restriction, with a minimum follow up of 1 year, outcomes and complications adequately reported. The studies had quality assessed using “Grading of Recommendations, Assessment, Development and Evaluations” (GRADE) framework [13]. The evaluated outcomes were surgical site infection, prosthesis infection, prosthesis revision, and removal. The outcomes were compared with time, techniques, prosthesis types, and diabetes mellitus presence.

The exclusion criteria were case reports, articles about surgical technique, and in vitro tests. All studies about transgender patients were excluded, as they assess a specific population and different surgical procedures. The studies focused on the quality of life without outcomes assessment, and those that stated to have no complications were excluded either. Considering infection incidence, studies that started with less than a hundred patients were excluded due to the risk of underestimation of complications.

Each study had data extracted including author, publication year, study design, penile prosthesis type, the number of patients, mean age, follow-up, infection, reoperation, prosthesis removal, or replacement. The data are presented as incidence ratio, with mean and standard deviation when available. The relation between diabetes mellitus and penile prosthesis infection is presented with a meta-analysis, and *odds ratio* calculated with Open Meta for Macintosh version 12.11.14. Significance was adopted as *p* < 0.05 and 95% confidence interval (95% CI).

## Results

### Literature overview

**Fig. 1:**
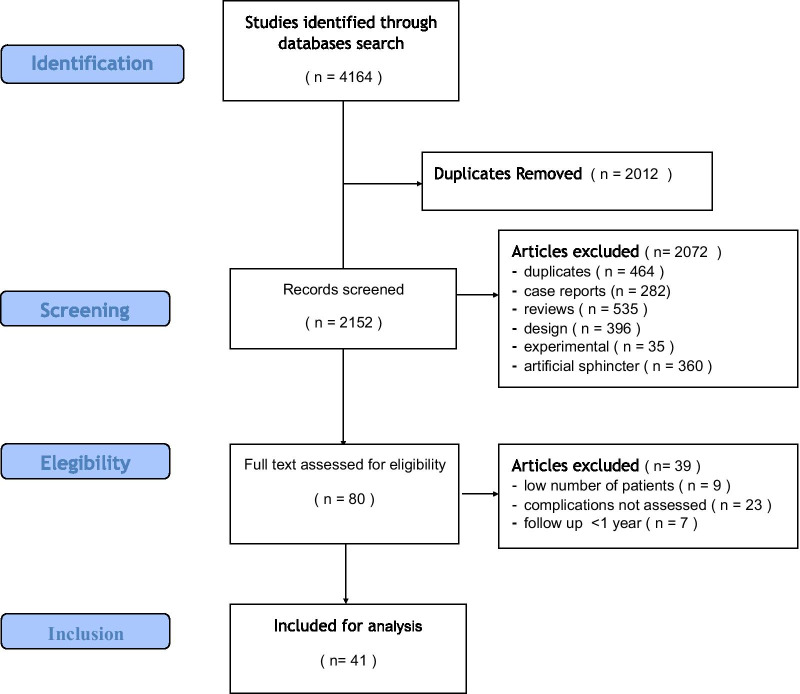
Study flow diagram. The diagram shows methodological steps of the systematic review

The research strategy was completed in January 2018 and turned out 4164 articles. After excluding 2012 duplicates, the 2152 articles had titles analyzed by two authors, who excluded yet 464 duplicates. The remaining had the abstract analyzed to exclude case reports, experimental studies, retrospective, and small series. In the end, 80 articles were fully assessed for eligibility, and 41 included in the analysis. The study selection is shown in a flow diagram (Fig. 1), while Table 1 presents the characteristics of the included studies and the infection rates.

**Table 1:** Included articles. The list of studies included as results of the systematic review, and infection rates

	Study	Year	Design	Level of evidence grade	Period	Prosthesis type	Patient *n*	Infection *n*	Infectionrate (%)
1	Carson et al. [34]	1983	Prospective	Low quality	1979–1982	Inflatable	100	1	1.00
2	Furlow et al. [35]	1987	Prospective	Low quality	1985–1987	Inflatable	120	1	0.83
3	Kabalin and Kessler [36]	1988	Prospective	Low quality	1975–1985	Scott reoperation	153	4	2.61
						Scott naive	264	5	1.89
4	Cumming and Pryor [37]	1991	Prospective	Low quality	1983–1987	Inflatable and malleable	280	32	11.43
5	Radomski and Herschorn [38]	1992	Prospective	Low quality	1979–1989	Inflatable and semi-rigid	269	6	2.23
6	Bishop et al. [15]	1992	Prospective	Low quality	1987–1988	Not sppecified	90	5	5.56
7	Goldstein et al. [39]	1993	Prospective	Low quality	1989–1991	Alfa 1—Mentor	112	3	2.68
8	Choi et al. [40]	1994	Retrospective	Low quality	1983–1993	Variable	295	3	1.02
9	Fein et al. [41]	1994	Prospective	Low quality	1988–1991	GFS II—Mentor	122	5	4.10
10	Wilson and Delk [16]	1995	Prospective	Low quality	1986–1993	Inflatable reoperation	428	43	10.05
						Inflatable naive	823	24	2.92
11	Holloway and Farah [42]	1997	Prospective	Low quality	1989–1994	Inflatable	145	3	2.07
12	Anafarta et al. [43]	1998	Prospective	Low quality	1989–1998	AMS Dynaflex	120	5	4.17
13	Wilson et al. [10]	1998	Prospective	Low quality	1994–1996	Inflatable	389	21	5.40
14	Garber and Marcus[44]	1998	Prospective	Low quality	7 years	Mentor A1 (3piece IPP)	360	6	1.67
15	Kabalin and Kessler [45]	1998	Prospective	Low quality	1975–1980	Scott; Small-carion	145	5	3.45
16	Montague et al. [46]	2001	Retrospective	Low quality	1986–1999	3 piece inflatable; database	491	10	2.04
17	Cakan et al. [47]	2003	Retrospective	Low quality	1993–2000	Malleable 2 piece	135	12	8.89
18	Ferguson and Cespedes [48]	2003	Prospective	Low quality	1992–1996	Malleable	94	1	1.06
19	Carson [17]	2004	Database	Low quality	2001–2003	AMS 700 InhibiZone™	2261	15	0.66
						AMS 700 no coating	1944	32	1.65
20	Wolter and Hellstrom [12]	2004	Database	Low quality	2002–2003	Titan coated	2357	25	1.06
						Alpha 1—no coating	482	10	2.07
21	Minervini et al. [49]	2005	Prospective	Low quality	1975–2000	Malleable—variable	504	40	7.94
22	Wilson et al. [23]	2007	Database	Low quality	2001–2004	AMS 700 InhibiZone™ naive	306	1	0.33
						AMS 700 InhibiZone™ reoperations	161	8	4.97
23	Kim et al. [50]	2010	Prospective	Low quality	1991–2009	AMS 700	397	8	2.02
24	DiBlasio et al. [51]	2010	Retrospective	Low quality	1997–2007	Inflatable	79	5	6.33
25	Carson et al. [19]	2011	Database	Low quality	2001–2008	AMS 700 no coating	3527	81	2.30
						AMS 700 inhibiZone™	34,556	408	1.18
26	Mulcahy and Carson [11]	2011	Database	Low quality	2001–2008	Inflatable coated	35,737	394	1.10
						Inflatable no coating	3268	82	2.51
27	Caire et al. [52]	2011	Retrospective	Low quality	2005–2007	Variable; reoperation	105	7	6.67
28	Dhabuwala et al. [20]	2011	Retrospective	Low quality	2002–2010	Titan; AMS InhibiZone™	497	10	2.01
29	Chung et al. [14]	2013	Prospective	Low quality	2006–2010	AMS 700; Titan (both coated)	138	3	2.17
30	Eid et al. [26]	2012	Prospective	Low quality	8.5y	AMS 700 InhibiZone™	704	14	1.99
						AMS 700 InhibiZone™ + “no touch”	1511	7	0.46
						AMS no coating	132	7	5.30
31	Omarbasha et al. [53]	2012	Retrospective	Low quality	2001–2011	Variable no coating	74	2	2.70
						InhibiZone™ and Titan	118	5	4.24
32	Henry et al. [54]	2012	Prospective	Low quality	2000–2007	Variable reoperation	214	12	5.61
33	Henry et al. [55]	2011	Prospective	Low quality	2000–2011	Variable; salvage surgeries	148	10	6.76
34	Chung et al. [2]	2012	Prospective	Low quality	1981–2010	Variable	955	14	1.47
35	Cohen and Eid [56]	2013	Prospective	Low quality	2003–2013	Variable coated reoperation	120	4	3.33
						Reoperation and “no touch”	283	1	0.35
36	Pozza et al. [57]	2015	Prospective	Low quality	1984–2013	Variable	500	15	3.00
37	Mohamed et al. [29]	2016	Retrospective	Low quality	2008–2015	Malleable	128	7	5.47
38	Chiang et al. [7]	2016	Prospective	Low quality	2004–2008	Variable	91	6	6.59
39	Antonini et al. [58]	2016	Prospective	Low quality	2011–2013	AMS 700 e Titan both coated	180	5	2.78
40	Katz and Love [59]	2017	Prospective	Low quality	2012–2015	Inflatable coated + “no touch”	150	1	0.67
41	Sevinc et al. [60]	2017	Prospective	Low quality	1998–2012	Malleable and inflatable	181	4	2.21

The literature about penile prosthesis relies on prospective cohorts, retrospective studies, and case series; the majority of studies do not present controls, or use historical data as controls. There is only one randomized trial available about the AMS 700 (*American Medical Systems, Minneapolis, MN, USA*) and the Titan (*Coloplast, Minneapolis, MN, USA*), presented in 2013 [14]. Both are inflatable and coated penile prosthesis, and were evaluated for satisfaction, curvature correction for Peyronie’s disease and mechanical survival. There were only 2 cases of infection from 138 patients with no report of group or time. The authors state that there was no statistical difference between groups, and the study was not designed to assess infection. Table 1 shows all the included studies with the infection incidence in each one.

### Diabetes mellitus and penile prosthesis infections

Diabetes mellitus is a well-established risk factor for several infections; however, the relation with penile prosthesis infection is still controversial. Diabetic patients are more susceptible to infections because of impaired defense mechanisms, including leukocyte dysfunction and impaired mobilization to the infection site due to angiopathy [10].

**Fig. 2:**
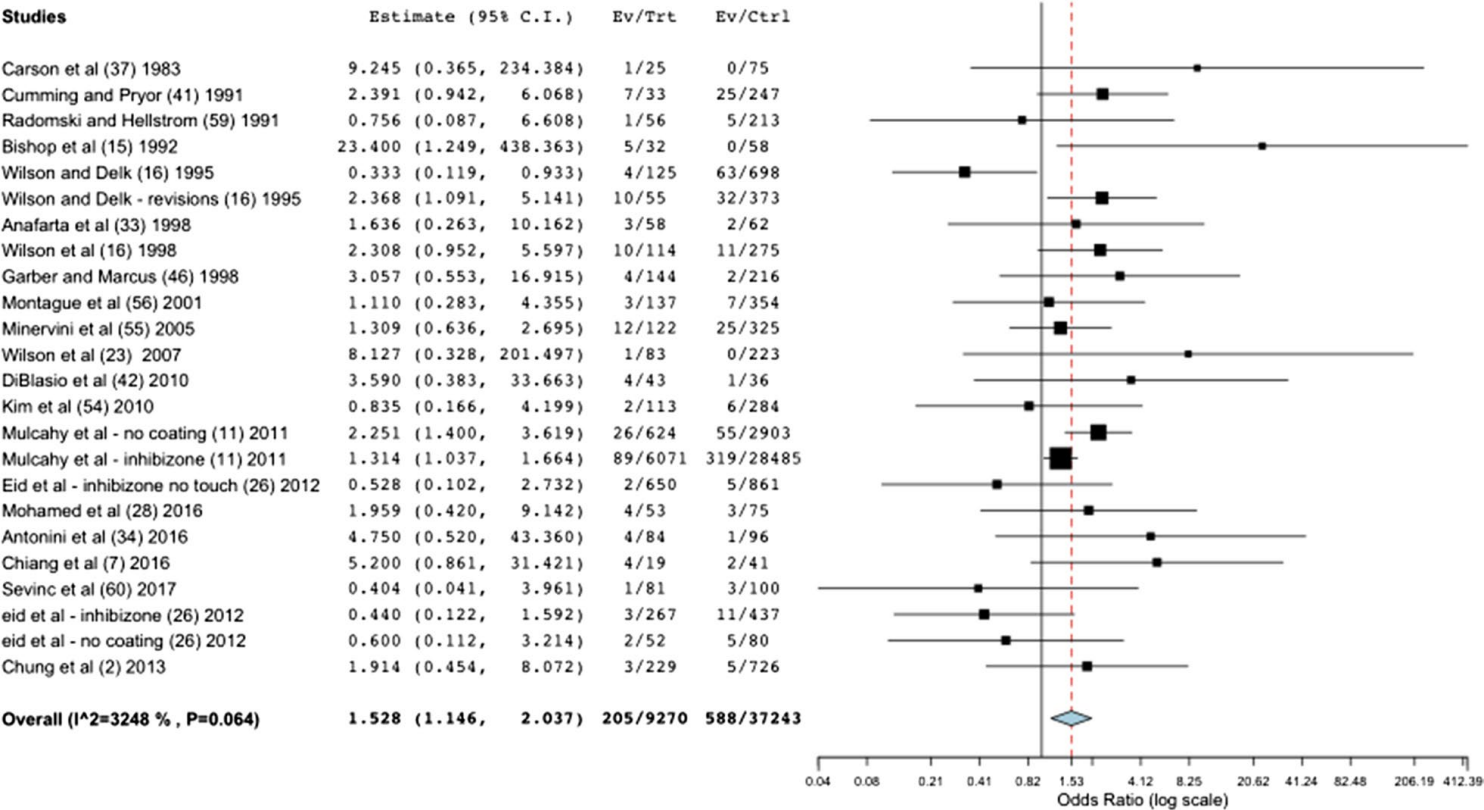
Forest plot diagram showing articles included in meta-analysis. The overall result shows that diabetic patients have more infections of penile prosthesis than non-diabetics, with an *odds ratio* of 1.53 (95% CI 1.15–2.04; *p* = 0.004)

We summarized the available evidence about penile prosthesis infection and DM in a meta-analysis, including 9041 diabetic patients and 36,517 non-diabetics. The meta-analysis shows that DM increases the incidence of penile prosthesis infection with an *odds ratio *of 1.53 (95% CI 1.15–2.04; *p* = 0.004), as shown in Fig. 2.

Fallon and Ghanen reported a three-fold higher risk of infection in diabetic patients, while Bishop et al. [15] suggested glycosylated hemoglobin as an infection predictor in the early 1990s. On the other hand, Wilson and Delk [16] found no relation between diabetes mellitus and infection in a retrospective study with 823 patients. However,  three years later, the same authors presented a prospective study with 114 diabetic patients and 275 non-diabetics and found a trend toward more infection in the diabetic group (8.8% vs. 4%; *p* = 0.06) [10]. Mulcahy and Carson [11], in a review of the manufacturer's database, including 31,341 men using a coated penile prosthesis, reported that the general revision rate was significantly higher in patients with diabetes: 1.72% versus 1.26% (*p* = 0.005).

### Decreasing infection strategies

The mean incidence of penile prosthesis infection decreased over time. Around the 1980s and 1990s, the incidence was about 8 to 11%, and in early 2000 it was 3 to 5%. The introduction of the coated prosthesis and technique improvements decreased the infection incidence to a current rate of about 0.3 to 2.7%. The incidence of infection in the included studies is shown in Table 1.

In 2000, the American Medical Systems (AMS) introduced a prosthesis coated with the InhibiZone™, which consists of an antibiotic coating using minocycline and rifampicin that elutes in tissues around the device and inhibit the bacterial growth. In 2004, Carson described results from the manufacturer's database, showing 0.28% rate of infection in prosthesis with InhibiZone™, while regular uncoated ones had 1.59% at 60 days after surgery (*p* 0.003). At 6 months, the incidence was 0.68% in the coated group and 1.61% in the control one (*p* 0.005) [17].

In 2002 Mentor (now Coloplast) introduced the Titan, which has a hydrophilic coating that reduces bacterial adherence and can diffuse antibiotics when immersed into an antibiotic solution during surgery [18]. In 2004, Wolter and Hellstrom published data about infection from Mentor’s database and FDA explantation reports. At 1 year follow-up, the infection rate in Titan prosthesis implants was 1.06% (25/2357), while in non-coated prosthesis it was 2.07% (10/482) (*p* 0.033) [12].

In 2011, Carson et al. published an extensive manufacturer's database review, including more than 39 thousand implants, with 90% of them with InhibiZone™; implanted between 2001 and 2008 and followed up to 7.7 years. They found that the revision rate for all causes (not only due to infection) was significantly lower on coated implants (6.7% vs. 12.5%, log-rank *p* = 0.002) [19]. Dhabuwala et al. compared the Titan immersed in rifampicin 10 mg/ml and gentamicin 1 mg/ml or vancomycin and gentamicin to AMS with InhibiZone™. There was no difference in infection rates between InhibiZone™ (1/77) and Titan with rifampicin and gentamicin (0/81). However, the vancomicyn + gentamicin group had 4.4% of infection (8/181), which was significantly higher than the other two groups (*p* < 0.05) [20]. Coated implants also had better results than regular ones regarding infection when used in reoperations [21–23]*.*

The concept of “center of excellence” is widely used for heart and oncologic surgeries, based on the evidence that surgeons with a high volume of a specific surgery trend to have superior outcomes. In 2009, Henry et al. introduced this concept to the urological field, comparing the results of penile prosthesis implants of a high volume urologist with ten general urologists. The single urologist had more than 50 cases per year, had shorter operative time (34 min versus 94 min, *p* < 0.0001), and eight-fold fewer reoperations (*p* 0.028). The concept was adopted and included as a recommended strategy to reduce infection [24, 25].

The most recent strategy was a technical improvement, presented by Eid in 2011, called the “*no-touch*” technique. It includes an antibiotic coated drape over the skin to reduce contact of hands and materials with the patient's skin [26]. In 2012, the same authors achieved an infection incidence of 0.4% using coated prosthesis and the “*no-touch*” technique [26].

## Discussion

This review presents essential information from a wide variety of available articles in ten databases and brings contemporary data about penile prosthesis infections. It summarizes device and technique improvements that contributed to reduction of infection and reoperations. Although penile prosthesis infection has decreased over the last decades, it is still a feared complication once it leads to reoperation, loss of function, and increases costs [8, 27].

For the first time, we present a meta-analysis about diabetes mellitus role in penile prosthesis infection, which brings light to a long controversy. The meta-analysis suggests that DM is related to a higher risk of penile prosthesis infection, with an *odds ratio* of 1.53. There is a considerable heterogeneity, which comes from the different studies' designs, and significant disparity in the number of subjects. The results at both sides of the forest plot show the controversy in the literature.

While older studies, from the 1990s, started to suggest the higher infection rates in diabetic patients, subsequent studies did not confirm it [28]. However, it is crucial to notice that most of the studies were not designed to evaluate DM properly, and most of them lack information about diabetes treatments and glucose control. For example, Mohamed et al*.* [29] reported that all patients in his study had glycosylated hemoglobin inferior to 7.0%. Thus, one may consider the contemporary practice to achieve good glycemic control before elective surgeries, and the lack of information about glycemic control on the databases. That may limit the evaluation of the glycosylated hemoglobin (Hb1Ac) role in most studies and contribute to the controversy regarding diabetes mellitus relation with infection.

In this scenario, it is essential to look at a prospective study designed to predict the importance of Hb1Ac levels at penile prosthesis infection. Habous et al. [30] recently analyzed 902 patients, who received different types of penile prosthesis, and found that Hb1Ac was significantly related to a higher incidence of infection. They had 80 implants with infection, which means an infection rate of 8.9%. The mean Hb1Ac in patients with infection was 9.5%, and it was significantly higher than in patients with no infection, with a mean Hb1Ac of 7.8% (*p* < 0.001). They constructed a ROC curve and proposed the Hb1Ac level of 8.5% as the threshold to predict infection with 80% sensitivity and 65% specificity.

Li et al. [6] also in 2018 reported diabetes mellitus, HIV, and Charles Comorbidity Index as factors associated with prosthesis removal. On the other hand, a recent retrospective study performed by Canguven et al. [31] included 300 patients and had only 2 cases of prosthesis infection, and both of them on non-diabetic patients. A superficial comparison could easily trick with the conflicting results, but one needs caution to interpret the studies' designs. A retrospective cohort may present biases, mainly due to the lack of information provided by patient charts or data loss, which invariably interfere with the results. The recent studies focused on penile prosthesis complications confirm our meta-analysis finding.

Our review is limited by the quality of the available evidence, the lack of controls, and studies based on the manufacturer’s database and FDA reports, which may have standardization and selection biases. However, it is crucial to consider the low incidence of infection, which requires a very high number of patients to show a decrease of incidence. Considering a baseline infection rate of 3%, it is estimated that a prospective study would require about 3 thousand patients to show a 50% reduction on infection rate or 34 thousand patients to show a 25% reduction [32]. It is also difficult to propose a trial to compare coated and uncoated implants when the available evidence suggest the superiority of the coated ones, which could bring ethical issues to the trial [33].

This is the most extensive review about penile prosthesis infection to our knowledge, including references from 10 databases, which brings information from the current scenario of penile prosthesis infection and gathers enough data to perform the first meta-analysis about the role of DM in penile prosthesis infection. The results encourage further studies focused on diabetic patients, which will be interesting to evaluate glycosylated hemoglobin levels, treatments in use, and the time elapsed from DM diagnosis to surgery.

## Conclusions

Penile prosthesis infection decreased in the last decades due to several improvements in materials and techniques. It remains a significant complication, and the meta-analysis indicates that diabetes mellitus is related to a higher risk of penile prosthesis infection.

## Data Availability

All data is fully provided. The subject of research was previous studies since it is a systematic review. All the studies are listed in a table and the full references are provided.
